# Confounding effect of blood volume and body mass index on blood neurofilament light chain levels

**DOI:** 10.1002/acn3.50972

**Published:** 2020-01-01

**Authors:** Ali Manouchehrinia, Fredrik Piehl, Jan Hillert, Jens Kuhle, Lars Alfredsson, Tomas Olsson, Ingrid Kockum

**Affiliations:** ^1^ Department of Clinical Neuroscience Karolinska Institutet Stockholm Sweden; ^2^ Centre for Molecular Medicine Karolinska University Hospital Stockholm Sweden; ^3^ Departments of Medicine, Biomedicine, and Clinical Research Neurologic Clinic and Policlinic University Hospital Basel University of Basel Basel Switzerland; ^4^ Institute of Environmental Medicine Karolinska Institutet Stockholm Sweden

## Abstract

Blood Neurofilament light chain (NfL) has been suggested as a promising biomarker in several neurological conditions. Since blood NfL is the consequence of leaked NfL from the cerebrospinal fluid, differences in individuals’ Body Mass Index (BMI) or blood volume (BV) might affect its correlation to other biomarkers and disease outcomes. Here, we investigated the correlation between plasma NfL, BMI, and BV in 662 controls and 2,586 multiple sclerosis cases. We found a significant negative correlation between plasma NfL, BMI/BV in both groups. Our results highlight the potential confounding effect of BMI/BV on associations between blood NfL and disease outcomes.

## Introduction

Neurofilament light chain (NfL) proteins are part of the neuronal structure supporting the radial growth of axons and maintaining their size, shape, and caliber. As result, larger myelinated axons abundantly express NfL even in healthy individuals in an age‐dependent manner. Increased NfL levels in cases compared to age‐matched controls are present in the cerebrospinal fluid (CSF) across a range of conditions characterized by neuronal and axonal degeneration including multiple sclerosis, Alzheimer’s disease, amyotrophic lateral sclerosis, frontotemporal dementia, HIV‐associated dementia, etc.^1^, ^2^ The recently developed single molecule array can detect very low concentrations of leaked NfL (pg/mL) from CSF in blood. Serum and plasma NfL measures have been used as a biomarker of disease activity and treatment response in multiple sclerosis^3^; however, to what degree differences in individuals’ blood volume (BV) and body mass index (BMI) might affect the correlation between blood and CSF concentrations of NfL has not been investigated.

## Materials and Methods

Blood samples and self‐reported weight and height measures from population‐based controls and multiple sclerosis (MS) cases were collected, concurrently as part of the *Epidemiological Investigation of MS* (EIMS)^4^ and *Immunomodulation and MS Epidemiology* (IMSE) projects^5^ in Sweden. Concentrations of plasma NfL (pNfL) were determined with antibodies from UmanDiagnostics and the high‐sensitive Single Molecule Array (SimoaTM) NF‐light^®^ Advantage kit. Detailed information on the study participants and study procedure has been previously described.^6^, ^7^

BV was calculated separately for males and females based on weight and height using the Nadler et al. formula.^8^ The association between log pNfL, BV, and BMI in MS cases and controls were independently assessed using multiple linear regression models adjusted for sex and age at the time of sampling. As a comparison, we investigated the association between CSF NfL and weight and height and BMI in relapsing MS cases. We also investigated the associations between pNfL, CSF NfL, and a clinical outcome after controlling for the effect of BMI on pNfL level. Semipartial Pearson correlation between log CSF and plasma NfL was computed in 32 cases with sampling gap between CSF and plasma measures of less than 60 days. The association between pNfL and the Expanded Disability Status Scale (EDSS) score, measured at the time of sampling (±30 days), was estimated using median regression models given the non‐normal distribution of the EDSS score.

## Results

We included 662 population‐based controls and 2,586 MS cases. Median pNfL level was 7.52 pg/mL (interquartile range (IQR): 5.87–9.87) in controls and 11.68 pg/mL (IQR: 8.27–18.46) in cases. There was no significant differences in proportion of females (75.2% vs. 72.6%, *P* = 0.19), sampling age (40 vs. 40, *P* = 0.97) and BMI (23.79 vs. 24.10, *P* = 0.1) between cases and controls. However, BV was significantly lower in cases compared to controls (4.44 vs 4.51, *P* = 0.03).

**Figure 1:**
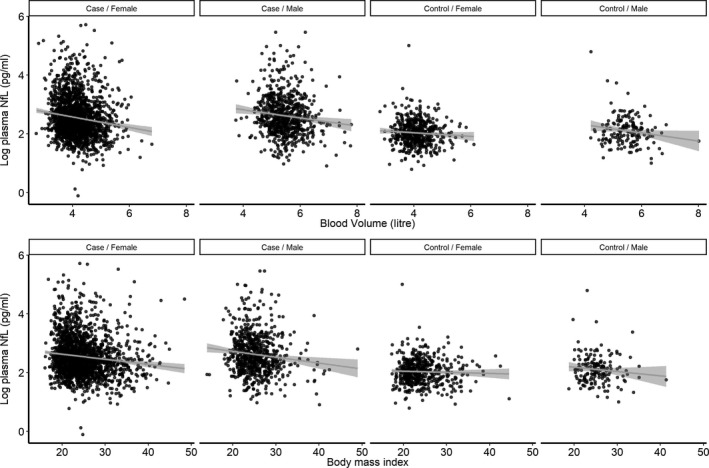
The association between plasma neurofilament light chain, blood volume, and body mass index in 662 population‐based controls and 2,586 MS cases stratified by sex.

**Figure 2:**
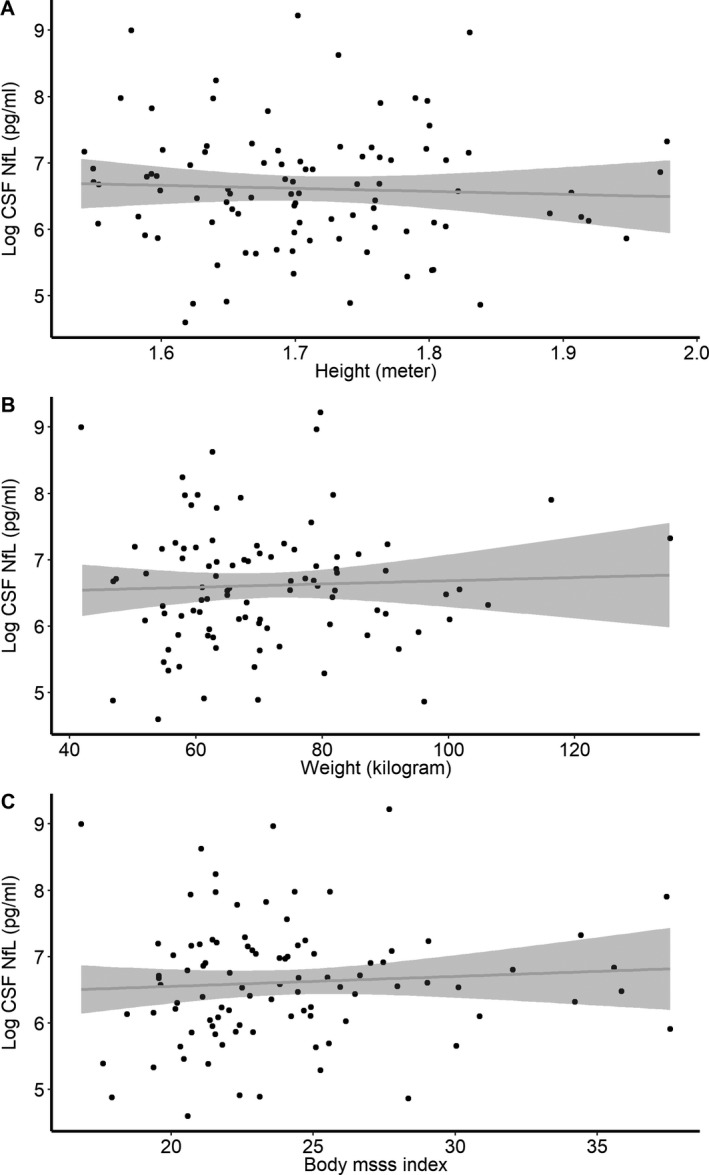
Association between log CSF neurofilament light chain level and weight (A), height (B), and body mass index (C). Data from 100 relapsing remitting multiple sclerosis cases reporting their height and weight within a year from CSF sampling. No significant association was found between weight, height and blood volume, and CSF NfL level after adjusting for sex and age at sampling.

There was a significant association between BMI and log pNfL after adjusting for sex and sampling age. Plasma NfL decreased by 0.02 (95% CI: −0.02 to −0.01, *P* < 0.001) in cases and −0.02 pg/mL (95%CI: −0.03 to −0.01, *P* < 0.001) per unit of BMI in controls. Similar association was seen between BV and log pNfL. Plasma NfL decreased by −0.15 pg/mL (95% confidence intervals (CI): −0.20 to −0.09, *P* < 0.001) and −0.17 pg/mL (95%CI: −0.22 to −0.12, *P* < 0.001) per liter increase in BV in controls and MS cases, respectively (Fig. 1). We did not observe any association between CSF NfL measures and cases’ height or weight or BMI (Fig. 2). The correlation between log CSF NfL and pNfL was improved by controlling for the effect of BMI. The correlation was increased from 0.80 (*P* < 0.001) to 0.85 (*P* < 0.001) after controlling for the effect of BMI on pNfL. In 1011 cases with available EDSS score at the time of sampling, a slight increase in the regression coefficient was observed after controlling for the effect of BMI. The regression coefficient increased from 0.51 (95%CI: 0.28–0.74) to 0.56 (95%CI: 0.34–0.79) after adjusting for BMI.

## Discussion

Plasma NfL concentrations have been shown to have a moderate correlation to different clinical and imaging outcomes in MS. The fact that we here see also a correlation with BMI and BV suggests that such correlations might change if corrections for BV or BMI (since both measures are driven by height and weight) are included. Also, from a generic point of view our findings underscore the potential importance of taking BV or BMI into account when analyzing neuronal biomarkers in blood.

## Author Contributions

AM conceptualized the study, analyzed, and interpreted data, wrote, and revised the manuscript. LA contributed to data (PI of EIMS), interpretation of data, and revising the manuscript. JH contributed to data (PI of GEMS), interpretation of data, and revising the manuscript. FP was involved in design of the study, interpretation of data, and writing the manuscript. TO contributed to data (PI of IMSE), interpretation of data, and writing the manuscript. JK was involved in the design of the study, management of data generation, interpretation of data, and writing the manuscript. IK was involved in the design of the study, interpretation of data, and writing the manuscript.

## Conflicts of Interest

AM is supported by the Margaretha af Ugglas foundation. FP has received research grants from Biogen, Genzyme, Merck KGaA and Novartis, and fees for serving as Chair of DMC in clinical trials with Parexel. JH has received honoraria for serving on advisory boards for Biogen, Sanofi‐Genzyme, and Novartis and speaker’s fees from Biogen, Novartis, Merck‐Serono, Bayer‐Schering, Teva, and Sanofi‐Genzyme. He has served as P.I. for projects, or received unrestricted research support from, Biogen, Merck, Novartis, and Sanofi‐Genzyme. JK served on scientific advisory boards for Novartis Pharmaceuticals, Merck, Biogen, Sanofi Genzyme, Roche, and Bayer; has received funding for travel and/or speaker honoraria from Biogen, Sanofi Genzyme, Novartis, Merck Serono, Roche, Teva, and the Swiss MS Society; and research support from Bayer, Biogen, Merck, Sanofi Genzyma, Novartis, Roche, the ECTRIMS Research Fellowship Programme, University of Basel, Swiss MS Society, Swiss National Research Foundation (320030_160221). LA has received lecture honoraria from Biogen and Teva. TO has received unrestricted MS research grants, and/or lecture advisory board honoraria from Biogen, Novartis, Sanofi, and Roche. IK is supported by the Horizon 2020 Multiple MS grant no 733161.
